# Graphene quantum dots‐based targeted nanoprobes detecting drug delivery, imaging, and enhanced chemotherapy of nasopharyngeal carcinoma

**DOI:** 10.1002/btm2.10270

**Published:** 2021-11-26

**Authors:** Chaosheng Yu, Zhen Long, Qianhui Qiu, Fang Liu, Yiming Xu, Tao Zhang, Rui Guo, Wen Zhong, Shuixian Huang, Shuaijun Chen

**Affiliations:** ^1^ Department of Otorhinolaryngology Head and Neck Surgery, Zhujiang Hospital Southern Medical University Guangzhou China; ^2^ Guangzhou Red Cross Hospital Jinan University Guangzhou China; ^3^ Department of Otorhinolaryngology head and neck Surgery The Sixth Affiliated Hospital of Sun Yat‐Sen University Guangzhou China; ^4^ Department of Otorhinolaryngology Head and Neck Surgery First Affiliated Hospital of Gannan Medical University Ganzhou China; ^5^ Key Laboratory of Biomaterials of Guangdong Higher Education Institutes, Guangdong Provincial Engineering and Technological Research Centre for Drug Carrier Development, Department of Biomedical Engineering Jinan University Guangzhou China; ^6^ Department of Otorhinolaryngology‐Head and Neck Surgery, Zhujiang Hospital Southern Medical University Guangzhou China; ^7^ Gongli Hospital of Shanghai Pudong New Area Shanghai China

**Keywords:** drug delivery, fluorescence resonance energy transfer, graphene quantum dots, nasopharyngeal cancer, tumor targeting

## Abstract

One of the main clinical treatments for advanced nasopharyngeal carcinoma is chemotherapy, but systemic administration can cause serious adverse reactions. New type of nanomaterial which can actively targeting, imaging, and treating nasopharyngeal carcinoma at the same time to enhance the effect of chemotherapy, meanwhile monitoring the intracellular drug release process at the level of single cancer cell was urgently needed. GE11, an EGFR antagonist peptide, was used to target nasopharyngeal carcinoma which has positive expression of EGFR on its nucleus. GE11‐modified graphene quantum dots (GQDs@GE11) were used as drug carriers for clinical chemotherapeutics cisplatin (CDDP) and doxorubicin (DOX). The emission spectrum of GQDs (460 nm) and the excitation spectrum of DOX (470 nm) have a good overlap, thus the transfer and release process of DOX can be sensitively detected by the fluorescence resonance energy transfer (FRET). CDDP was used to enhance the chemotherapy effect of nanoprobe, and the loading amount of DOX and CDDP on GQDs@GE11 nanoprobe were up to 67 and 50 mg/g, respectively. In vivo experiments have confirmed that GQDs@GE11/CDDP/DOX nanoprobe can be enriched to tumor site through specific targeting effect, and significantly inhibit tumor cell proliferation. This new type of targeted therapy fluorescent probe provides new ideas for the study of drug release process and the treatment of nasopharyngeal carcinoma.

## 1 INTRODUCTION

Nasopharyngeal carcinoma is a common malignant tumor of the head and neck,^1^, ^2^, ^3^ it has a deep focus and the clinical manifestations of nasopharyngeal carcinoma are not obvious. Therefore, once diagnosed, nasopharyngeal carcinoma usually reaches an intermediate or advanced stage. High‐intensity radiotherapy and chemotherapy can effectively control and kill tumor cells,^2^, ^4^ but due to lack of selectivity, normal tissues are also severely damaged, leading to many complications, which greatly affect the treatment effect and plan.^5^, ^6^ Therefore, there is an urgent need to develop a new treatment method with minimal side effects at low doses and the best antitumor effect for the treatment of nasopharyngeal carcinoma.^7^

Drug delivery platforms based on nano‐drugs have been widely studied and applied in the treatment of nasopharyngeal carcinoma.^1^, ^8^, ^9^ Among inorganic nanocarriers, graphene derivatives such as graphene oxide (GO) and graphene quantum dots (GQDs) are less toxic compared with other inorganic nanomaterials containing heavy metal ions,^10^ and have been used for tumor therapy research for many years.^11^, ^12^ The high drug loading capacity, excellent physiological stability and biocompatibility, strong photoluminescence, and ease of use of GQDs have also been proven, which makes them a promising nanocarrier for the delivery of anticancer drugs.^13^, ^14^, ^15^, ^16^ The importance of targeting delivery has been emphasized due to the systemic toxicity caused by nonspecific administration. Specific targeting to cancer tissues or cancer cells can significantly reduce the side effects of chemotherapy drugs.^17^ Nasopharyngeal carcinoma has positive expression of EGFR on the tumor cells nucleus.^18^ GE11, an EGFR antagonist peptide, is a potential targeted modification peptide for nasopharyngeal carcinoma.^19^

Fluorescence resonance energy transfer (FRET) is an interesting technique which can transmit photo‐excitation energy from a donor fluorophore to an acceptor fluorophore.^20^ FRET‐based fluorescence quenching and reproduction can be used for biological detection or monitoring of some dynamic processes, such as the delivery process of drugs.^21^ In recent years, people have studied the potential applications of GQDs in bioimaging and drug delivery.^21^, ^22^, ^23^ GQDs have been applied in biosensing owing to its incredible fluorescence characteristics (such as high brightness, long fluorescence lifetime, and photostability).^20^ It can be excited by a short‐wavelength, like ultra‐violent, and the emission wavelength is 460 nm, which is very close to the excitation wavelength of doxorubicin (DOX, 450 nm), thus making it possible to construct fluorescent probes based on FRET.

At the same time, DOX is also one of the most famous anticancer drugs in clinical practice. GQDs were also reported to have a high drug loading rate for DOX.^17^, ^18^ However, single‐drug loading platforms usually require large doses of drugs to achieve the desired therapeutic effect, and excessive drugs can also cause systemic side effects. Therefore, the development of intelligent therapeutic nanoplatform with multiple synergistic antitumor drugs will be of great significance.^24^, ^25^ Cisplatin (CDDP) is a cell cycle nonspecific drug and has therapeutic effects on many tumors, including nasopharyngeal carcinoma.^26^ Nanoplatform combine DOX and CDDP will have enhanced therapeutic effects and low side effects at the same time.

**SCHEME 1:**
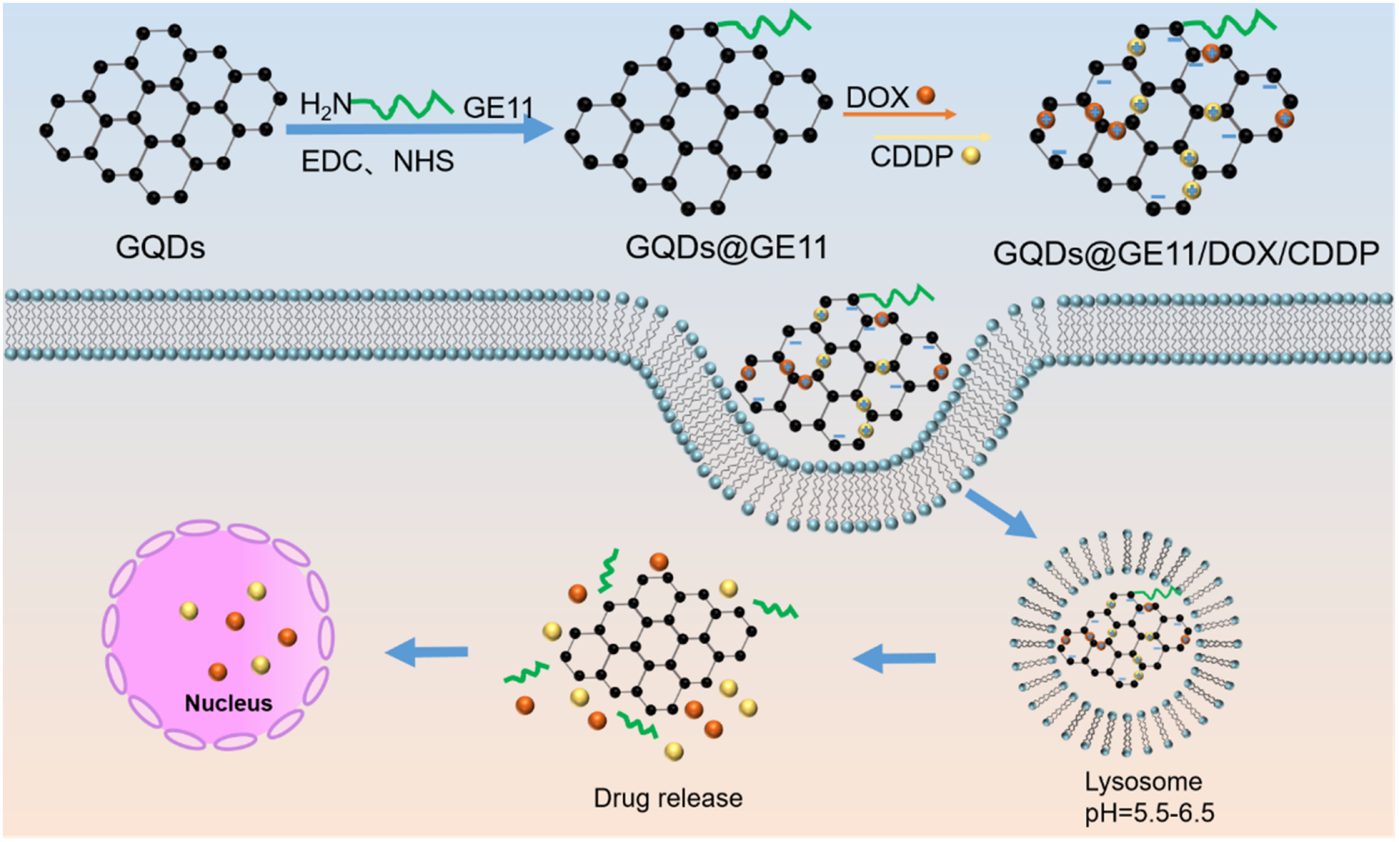
Transport DOX and CDDP to cell by the GQD@GE11/DOX/CDDP complex for tumor therapy

Here, we proposed a noncytotoxic, targeted, GQD‐based nanoprobe with dual functions of drug delivery and cell imaging. GQDs were modified with the targeting polypeptide GE11 to target EGFR which highly expressed on the nucleus of nasopharyngeal carcinoma.^17^, ^27^, ^28^, ^29^ GQDs nanoprobe had good biocompatibility and high drug loading capacity that would be a potential drug carrier for cancer treatment. DOX and CDDP, the two most widely used chemotherapeutics in clinical practice,^30^, ^31^ were loaded in the GQDs@GE11 nanoprobe to construct the anticancer nanoprobe GQDs@GE11/DOX/CDDP. Due to the inherent fluorescence imaging capabilities of GQDs and DOX, we also built a FRET system based on GQDs and DOX to study the cellular delivery and release of drugs. The co‐delivery system showed excellent DOX and CDDP delivery capabilities to CNE‐2 cells and tumors, and its combined antitumor effect was much better than using only DOX or CDDP treatment, indicating that it had potential applications in nasopharyngeal carcinoma therapy and visualization of intracellular drug uptake behavior (Scheme 1).

## 2 EXPERIMENTAL SECTION

### 2.1 Materials

All chemical reagents were purchased and used without further purification. Graphite powder, sulfuric acid, nitric acid, hydrogen peroxide, and sodium nitrate were purchased from Sinopharm Group Chemical Reagent Co., Ltd. (Guangzhou, China) Cisplatin and doxorubicin were purchased from Aladdin (Shanghai, China). 1‐ethyl‐3‐(3‐dimethylaminopropyl) carbodiimide hydrochloride (EDC) and N‐hydroxysuccinimide (NHS) were bought from Macklin Biochemical Technology Co., Ltd (Shanghai, China). RPMI 1640 medium, fetal bovine serum, trypsin, penicillin–streptomycin, and phosphate‐buffered saline were obtained from Gibco (California, USA). Cell counting kit‐8 (CCK‐8) was purchased from Beyotime Biotechnology (Shanghai, China). Annexin VPE apoptosis kit was bought from Becton, Dickinson, and Company (State of New Jersey, USA). GE11 peptide was synthesized by Sangon Biotech Co., Ltd (Shanghai, China). CNE‐2 (Nasopharyngeal carcinoma cell line) was supplied from Southern Medical University in Guangzhou.

### 2.2 Synthesis of GO

GO was synthesized through modified Hummer's method.^32^ The graphite powder was pre‐oxidated by the following process: 4.0 g of graphite powder was dissolved in 15 ml of H_2_SO_4_ solution containing 5.0 g K_2_S_2_O_8_ and 5.0 g P_2_O_5_ under 80°C. Then the dark mixture was separated and cooled overnight at room temperature. Finally, ultrapure water was used to diluted the mixture until neutral. As for the oxidization process, pre‐oxidized graphite was dissolved in 90 ml of cold H_2_SO_4_ solution containing 2.0 g of NaNO_3_. Subsequently, 12.0 g of KMnO_4_ was slowly added to the mixture and the reaction was stirred at 40°C for 8 h. After that, 200 ml of deionized water was added to the above solution and heated up to 90°C for 15 min. The oxidization process was stopped by adding a large amount of deionized water and 30% H_2_O_2_ solution. The final product GO was obtained by filtering and then washed with dilute HCl solution and deionized water for three times.

### 2.3 Synthesis of GQDs

GQDs was obtained by ultrasonic peeling.^33^ GO was dispersed in N, N‐dimethylformamide (DMF) at a concentration of 20 mg/ml, then the mixture was sonicated for 30 min (120 W, 100 kHz). After that, the mixed solution was transferred to a 40 ml autoclave lined with Teflon and heated at 200°C for 4 h. Then the container was cooled to 25°C with water and the black precipitate was collected, washed with water, and resuspended in PBS for later use.

### 2.4 Synthesis of GQDs@GE11

The coupling of GQDs and GE11 was achieved by amide reaction. Specifically, 24 mg of EDC and 24 mg of NHS were added to 3 ml of GQDs solution (1 mg/ml) and stirred for 4 h. Then, 10 mg of GE11 was added dropwise to the above solution and reacted at room temperature overnight. Finally, the mixture was dialyzed against deionized water (MWCO = 500 Da) for 72 h to remove impurities to obtain GQDs@GE11 solution.

### 2.5 Loading of CDDP and DOX on GQDs@GE11

For the preparation of DOX and CDDP co‐loaded complexes, DOX was firstly added to GQDs solution, and then CDDP was mixed. In short, 5 mg of DOX·HCl and 10 mg of GQDs@GE11 were dissolved in distilled water, and then 5 μl of triethylamine solution was added to the mixture and the reaction was stirred for 2 h to neutralize hydrochloric acid. Finally, 2 ml of cisplatin (2 mg/ml, DMSO) solution was added to the mixed system, and the reaction was stirred overnight. After that, the solution was dialyzed and lyophilized to obtain the GQDs@GE11/CDDP/DOX complex. The loads of DOX and CDDP in the GQDs@GE11/CDDP/DOX complex were determined by ultraviolet–visible spectrophotometry and high‐performance liquid chromatography, respectively.

### 2.6 Characterization of nanoprobe

The shape, size, and morphology of the synthesized nanoprobe were studied with transmission electron microscopy (JEOL TEM‐1210, USA) at 120 kV and Zetasizer Nano ZS (Malvern, UK) apparatus. Fourier transform infrared spectrum of all samples were collected in a PerkinElmer Spectrum 100 FT‐IR spectrometer (PerkinElmer Inc., USA) under the transmittance mode with KBr plates. Excitation and emission spectra of samples were recorded using a FLS920P Edinburgh Analytical Instrument. Confocal optical micrographs were analyzed by performing confocal laser scanning microscope (CLSM, Leica SP8, Nikon, Japan).

### 2.7 In vitro drug release

The GQDs/DOX/CDDP dispersion was placed in a dialysis bag (MWCO = 1 kDa), then immersed in a release container containing PBS (0.1 M, pH 5.5) and stirred at 37°C in the dark. Two milliliter of sustained release buffer was collected from each container at different time intervals and replaced with 2 ml of fresh PBS. In order to measure the amount of drug released in each time interval, the collected samples were analyzed by high‐performance liquid chromatography and UV–vis spectroscopy.

### 2.8 Cytotoxicity of GQDs@GE11

The cytotoxicity of GQDs and GQDs@GE11 was measured on CNE‐2 cells using the CCK‐8 assay. Briefly, the CNE‐2 cells were seeded in 96‐well plates with cell density of 5 × 10^3^ cells per well and cultured for 12 h. The cells were then incubated with fresh cell medium containing GQDs or GQDs@GE11 with concentrations ranging from 0 to 100 μg/ml and incubated for 24 h. After treatment, cells were washed with PBS and added with fresh cell medium containing 10% CCK‐8 to all wells. Finally, the absorbance at 450 nm was tested by a microplate reader (Thermo Scientific, USA). CNE‐2 cells incubated with RPMI 1640 medium were used as control groups.

### 2.9 GE11 targeting ability assay

Cellular uptake of GQDs@GE11/DOX/CDDP complex with and without GE11 functionalization was analyzed to confirm the targeting ability of GE11. In detail, CNE‐2 cells were seeded in 24‐well plate with a density of 5 × 10^4^ cells/well and incubated in a 37°C humidified incubator (5% CO_2_) for 12 h. Then, GQDs or GQDs@GE11 complex (GQD:50 μg/ml) was added to treat the cells and incubated for different time. After each interval (0.5, 2, 4, and 8 h), cells in each group were washed by PBS, trypsinized, centrifuged, and resuspended in 200 μl PBS in an Eppendorf tube. Finally, samples were measured using flow cytometry and the corresponding fluorescence intensity was quantified by Flow Jo 7.6.1 software.

### 2.10 In vitro cellular fluorescence imaging

The cellular uptake of GQDs@GE11/DOX/CDDP by CNE‐2 cells was quantified using CLSM measurements. In detail, CNE‐2 cells were seeded in 2 cm confocal microscopy dish at a density of 2 × 10^5^ cells per well and incubated for 12 h. Then, cells were treated with GQDs@GE11/DOX/CDDP (50 μg/ml) and incubated for different time. After each interval (0.5, 4, and 8 h), the cells were washed with PBS. Finally, the cells were detected at various time points with the same optical conditions using confocal microscopy for the following channels: GQDs channel (Ex: 405 nm; Em: 475–505 nm), DOX channel (Ex: 485 nm; Em: 585–615 nm), DOX FRET channel (Ex: 405 nm; Em: 585–615 nm).

### 2.11 In vitro cell proliferation inhibition

In order to measure the effect of co‐administration of GQDs@GE11/DOX/CDDP on CNE‐2 cell survival rate, CCK‐8 assay was performed. Briefly, CNE‐2 cells were planted into 96‐well plate at a density of 1 × 10^4^ cell per well. After overnight, the cells were exposed to GQDs@GE11/DOX/CDDP with drug concentrations for 24 h. Then, 100 μl of CCK‐8 solution (10%) was added to each well and incubated at 37°C in 5% CO_2_ atmosphere for 1 h. Next, absorbance of each sample was measured at 450 nm using a microplate reader.

### 2.12 Apoptosis assay

CNE‐2 cells were plated in 24‐well plate with a density of 5 × 10^4^ cells per well and incubated for 24 h. Subsequently, the cells were treated with different formulations with a CDDP concentration of 16 μg/ml. Cells without any treatment were used as control. Then, all cells were collected and resuspended in 500 μl PBS. After that, all cells were stained with PI and Annexin V‐fluorescein isothiocyanate V (FITC) containing binding buffer for 15 min, and finally detected by flow cytometry (Becton Dickinson, San Jose, California, USA).

### 2.13 Tumor inhibition assay

Twenty female Balb/C nude mice (6 weeks old, 18 g body weight) were purchased from Southern Medical University. All animal experimental protocols have been approved by the Animal Protection and Use Committee of Southern Medical University. A mouse nasopharyngeal carcinoma tumor model was established by subcutaneous injection of CNE‐2 cells (0.1 ml, 1.5 × 10^6^ cells). When the tumor grew up to about 100 mm^3^, tumor‐bearing mice were randomly divided into five treatment groups: PBS control, GQDs@GE11/DOX, GQDs@GE11/CDDP, GQDs/DOX/CDDP, and GQDs@GE11/DOX/CDDP (DOX: 2 mg/kg, CDDP: 2 mg/kg). Each experimental group included three mice and injected via tail vein every 2 days. The tumor volume was tested with an electronic caliper and calculated as: 1/2 × shortest diameter^2^ × longest diameter. At the same time, the weight of the mice was recorded. After seven treatments, the tumors were collected and weighed.

### 2.14 Histologic and immunohistochemical analysis

The mice of all groups were killed after the treatment. The tumors were removed, fixed in 4% paraformaldehyde for 24 h, embedded in paraffin, and cryosectioned into 4 μm slices. Then, the sections were stained with hematoxylin and eosin (H&E). For immunohistochemical analysis, the level of tumor apoptosis was evaluated by the terminal deoxynucleotidyl transferase deoxyuridine triphosphate nick end labeling (TUNEL) assay. In addition, the expression of Ki67 in tumor sections was also detected.

### 2.15 In vivo fluorescence imaging

In order to explore GQDs@GE11/DOX/CDDP in vivo fluorescence imaging and tumor enrichment. The 100 μl GQDs@GE11/DOX/CDDP (DOX:2 mg/kg) was intravenously injected, and the fluorescence images of the mice were recorded at 0.5, 1, 3, 5, and 8 h intervals using the in vivo FX Pro device (Brooker, USA). After 8 h, the mice were sacrificed and the fluorescence images of the main organs (heart, liver, spleen, lung, and kidney) and tumor tissue were captured. Take GQDs/DOX/CDDP complex without GE11 targeting peptide as control.

### 2.16 Biocompatibility evaluation

#### 2.16.1 In vitro hemolysis assay

The hemolysis rate was measured according to the previously reported method. In short, 2 ml of GQDs@GE11solution (0.01, 0.1, and 0.2 mg/ml) was added to 0.2 ml of 16% red blood cell (RBC) suspension and incubated for different time. After that, the hemolysis rate at different time points was tested at 540 nm by a microplate reader. The distilled water and PBS were used as positive control group and negative control group, respectively. Hemolysis ratio was calculated according to the following formula:

$$\text{hemolysis}\;\left(\%\right)=\left(M_{0}-M_{2}\right)/\left(M_{1}-M_{2}\right)\times 100$$

where M_0_ is the absorbance of the test sample, M_1_ is the absorbance of the negative control, and M_2_ is the absorbance of the positive control.

#### 2.16.2 Morphology of RBCs

Fresh whole blood was centrifuged at 1000×*g* for 5 min to obtain RBC. The RBC was incubated with GQDs@GE11 solutions of different concentrations for 10 min, washed with PBS, and then fixed with 4% paraformaldehyde for more than 1 h. The fixed RBC was deposited on a glass slide, dehydrated with 70%, 85%, 95%, and 100% (v/v) ethanol for 10 min, and then dried in air. The dried RBC was coated with gold and observed with a scanning electron microscope (LEO 1530 VP, Philips, Amsterdam, the Netherlands).

#### 2.16.3 Histological analysis

After the mice were euthanized, the heart, liver, spleen, lung, kidney, and other major organs were taken and immersed in tissue fixative. The sections were stained with H&E and imaged with fluorescence microscope.

#### 2.16.4 Blood chemistry assay

After 14 days treatment, the mouse blood was collected, centrifuged at 3000 rpm for 5 min, and the serum in the supernatant was collected. Then detect activated partial thromboplastin time (APTT) and prothrombin time (PT).

### 2.17 Statistical analysis

All data were represented as mean ± SD and each experiment was performed in triplicate for three separate experiments. Data were analyzed using one‐way analysis of variance (ANOVA) followed by Tukey's post hoc test or unpaired *t*‐test in Graphpad Prism Version 7 software. The statistical significance was *p < 0.05, **p < 0.01, and ***p < 0.001.

## 3 RESULTS AND DISCUSSION

### 3.1 Synthesis and characterization of nanoprobes

**FIGURE 1:**
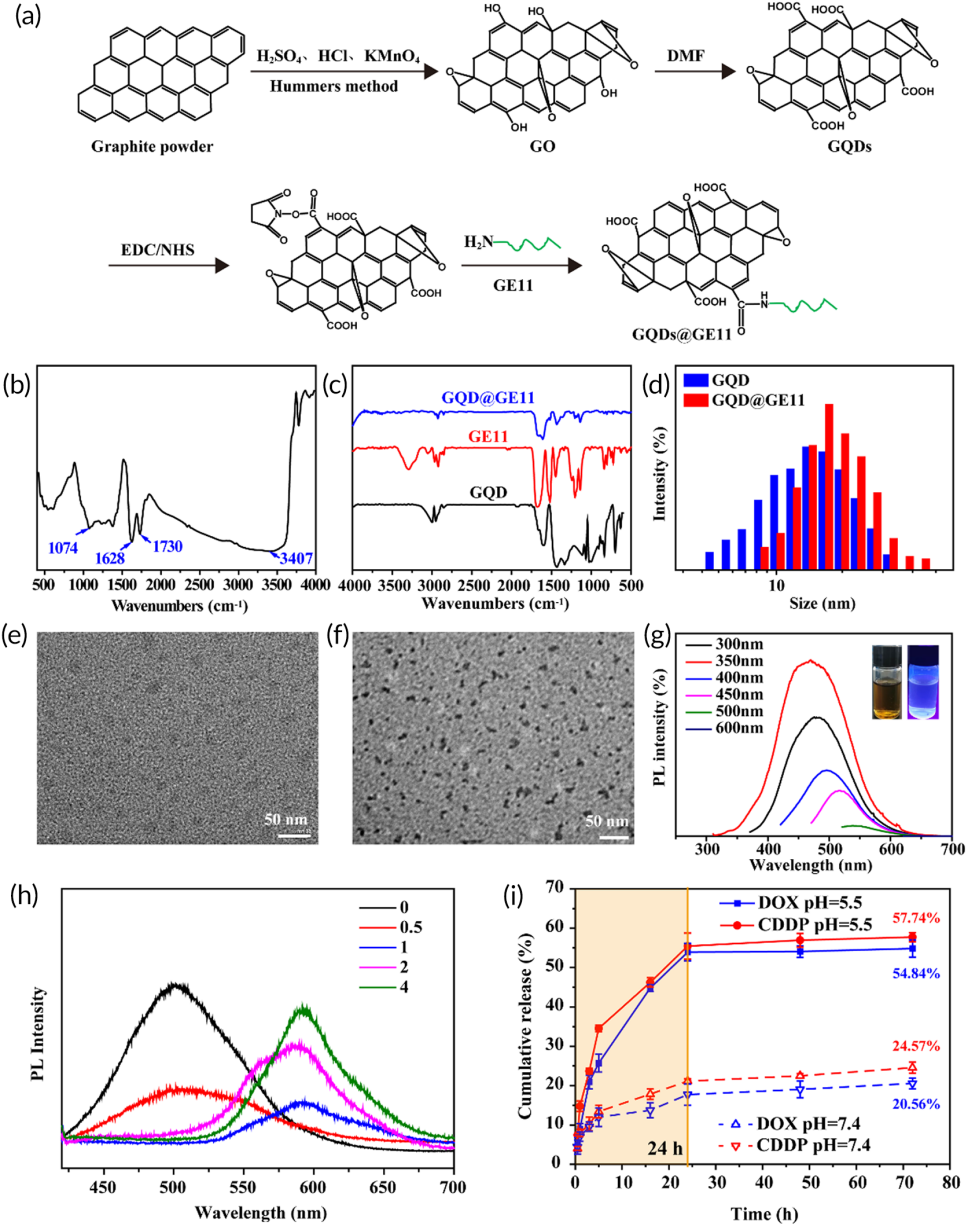
Characterizations of GQDs@GE11. (a) Schematic diagram of GQDs@GE11. (b) FTIR spectra of GO. (c) FTIR spectra of GQDs@GE11, GQDs and GE11. (d) Size distribution of GQDs and GQDs@GE11. (e) TEM image of GQDs. (f) TEM image of GQDs@GE11. (g) PL spectra of GQDs solutions under different excitation wavelengths. Inset: Aqueous dispersion photograph of GQDs under visible light (left) and UV at 365 nm (right). (h) PL spectra of GQDs@GE11/DOX/CDDP with increasing molar ratio of DOX (from 0 to 4). (i) Cumulative DOX and CDDP release (%) from GQDs@GE11/DOX/CDDP in PBS buffer (pH = 5.5 and pH = 7.4) values at different time intervals

The nanoprobes preparation was shown in Figure 1a. GO nanosheets were firstly obtained by the modified Hummers method from natural graphite powder. Then the GQDs were obtained and modified to obtain the GQDs@GE11/DOX/CDDP nanoprobe. GO had absorption peaks at 3407 and 1730 cm^−1^, and the absorption peak at 1628 cm^−1^ belonged to the bending vibration absorption peak of C—OH bond^34^ (Figure 1b). Figure 1c showed the FTIR spectra of nanoprobes. Due to the C—OH stretching vibration of the ‐COOH group, the infrared spectrum of GQDs had a peak at 1418 cm^−1^. Other peaks are observed at 1618 cm^−1^ due to the stretching vibration of the C=C bond. A value of 1130 cm^−1^ was the hydroxyl stretching vibration peak of GE11, and 1676 cm^−1^ was the characteristic band of C=O in the amide bond, which further proved the successful coupling of the peptide GE11 and GQDs.

The average particle size of GQDs and GQDs@GE11 detected by dynamic light scattering (DLS) was 11.73 ± 3.24 nm (PDI = 0.17) and 14.23 ± 3.89 nm (PDI = 0.16), respectively (Figure 1d). Both GQDs and GQDs@GE11 had negative zeta potential, for GQDs was −42.67 mV, while GQDs@GE11 was 26.17 mV. Transmission electron microscope (TEM) images of GQDs and GQDs@GE11 all showed a regular spherical shape and the diameters of GQDs and GQDs@GE11 were 2.86 ± 0.44 and 6.5 ± 1.5 nm, respectively. (Figure 1e, f). The increased diameter of GQDs@GE11 nanoprobe was owing to the modification of EGFR‐targeted peptide GE11.

The fluorescence properties of GQDs were evaluated by studying their photoluminescence (PL) behavior under different excitation wavelengths. The results showed that GQDs exhibit excitation‐dependent emission. When the excitation wavelength was increased from 300 nm to 600 nm, the PL intensity decreased significantly (Figure 1g). The strongest PL appeared at excitation wavelength of 350 nm centered at emission wavelength of 462 nm. As shown in Figure 1g (inset), the yellow GQDs aqueous solution emitted a strong blue PL under ultraviolet light. These results confirmed the application prospects of GQDs as cell imaging agents.

To further explore the energy resonance transfer system, a specific concentration of GQDs@GE11 (1 mg/ml) was hatched with DOX at a series of increasing molar ratios. When DOX was loaded onto the GQD surface through pi‐pi superposition interaction, the fluorescence signal of GQDs was quenched due to the energy transfer from GQDs to DOX.^35^, ^36^, ^37^ It was observed that the fluorescence signal of GQDs decreased successively, and the fluorescence signal of DOX continued to increase, indicating that the amount of DOX molecules adsorbed on the GQDs surface was increasing (Figure 1h). In contrast, the DOX emission peak at 580 nm was observed due to the FRET effect.

### 3.2 Drug release from GQDs@GE11

Due to the layered structure of GQDs, hydrophobic drugs DOX and CDDP could be encapsulated in their layers, resulting in high loading amount of DOX and CDDP as high as 67 and 50 mg/g, respectively. The GQDs has pH‐sensitive drug release property,^38^ after being immersed in PBS buffer (pH = 5.5) at 37 °C, DOX and CDDP encapsulated in GQDs@GE11 represented similar release profiles (Figure 1i). The release rates of DOX and CDDP after 6 h were 34.5% and 25.7%, respectively. Drug release rate gradually decreased and reached plateau after 24 h. The final drug release rates of DOX and CDDP were 54.8% and 57.7%, respectively. The DOX and CDDP encapsulated in GQDs@GE11 immersed in PBS (pH = 7.4) showed a significant lower release rate compared with acidic environment. Only 24.57% of CDDP and 20.56% DOX were released after 72 h. This indicates that the GQDs nanoprobes have pH‐sensitive drug release property, which is consistent with previous reports.^38^

### 3.3 GE11 targeting ability assay

**FIGURE 2:**
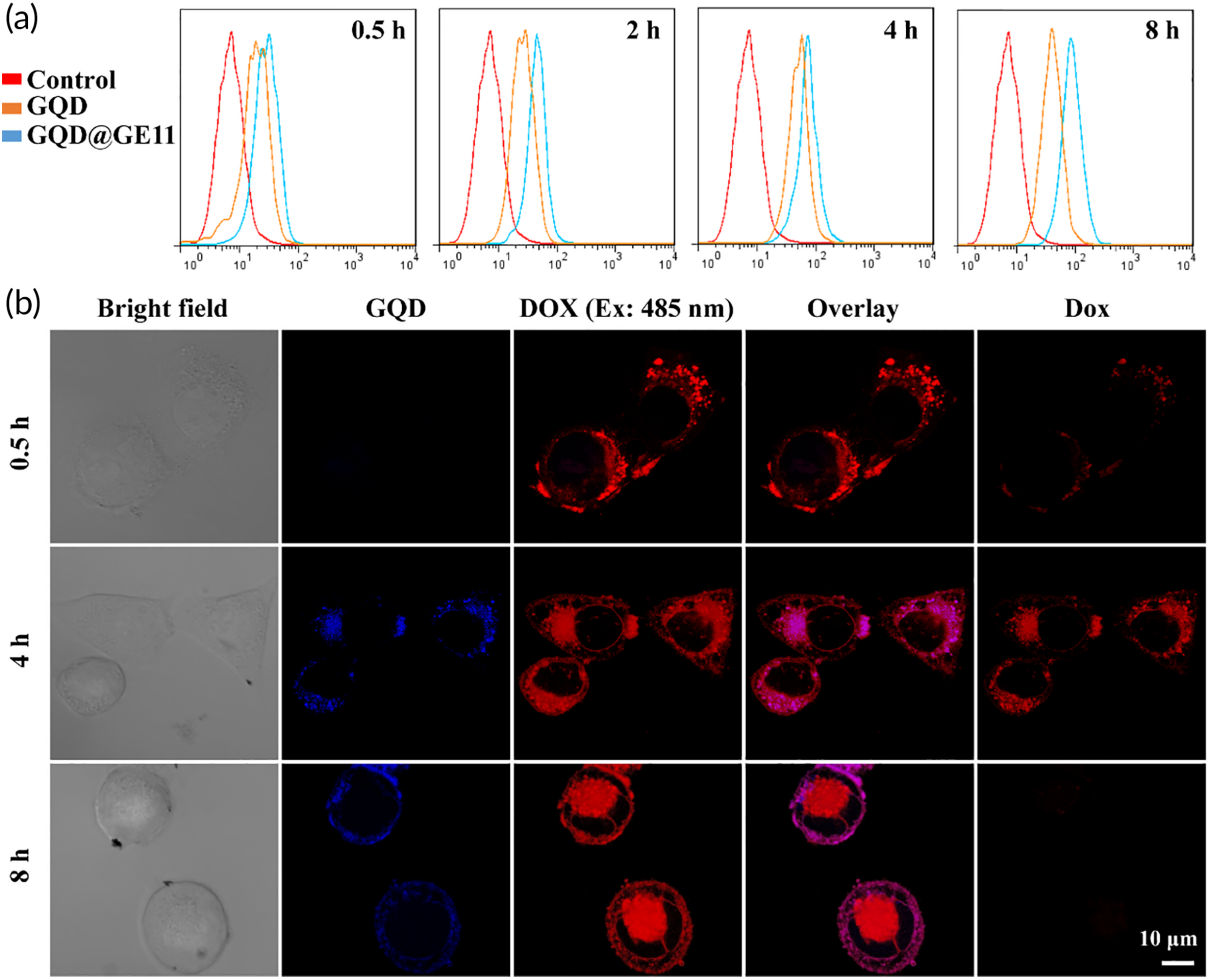
(a) Flow cytometry analysis of fluorescence peak in CNE‐2 cells incubated with GQDs or GQDs@GE11 for different time. (b) Fluorescence image of CNE‐2 cells treaded with GQDs@GE11/DOX/CDDP at 0.5, 4, and 8 h. GQDs channel (Ex: 405 nm; Em: 475–505 nm), DOX channel (Ex: 485 nm; Em: 585–615 nm), DOX FRET channel (Ex: 405 nm; Em: 585–615 nm)

Efficient uptake by in vivo cells was the key to achieve the biological performance of material design.^39^ The synthetic 12‐amino acid peptide GE11 (amino acid sequence: Y‐H‐W‐Y‐G‐Y‐T‐P‐Q‐N‐V‐I) has been proved to be an effective EGFR targeting peptide in vitro and in vivo^40^; therefore, it is one of the best choices for EGFR‐targeted diagnosis and drug delivery system design.^41^ In order to verify the targeting effect of GE11 on CNE‐2 cells, cellular uptake of GQDs and GQDs@GE11 were also compared by flow cytometry, and results are shown in Figure 2a. As expected, GQDs with GE11 function had the strongest fluorescence intensity at each time point, indicating that GQDs@GE11 absorbed by CNE‐2 cells increased significantly, which may be owing to the targeting effect of GE11 at EGFR receptor on CNE‐2 cells. This advantage of specific targeting at tumor cells may reduce the systemic toxicity of systemic administration.^42^

### 3.4 Intracellular imaging and drug release monitoring

Single fluorescence imaging method usually cannot visualize the release process of the drug on the nanocarrier. In this research, FRET imaging was used to visualize the GQDs@GE11/DOX/CDDP nanoprobe uptake in CNE‐2 cells and DOX release process. The emission spectrum of GQDs (460 nm) and the excitation spectrum of DOX (470 nm) have a good overlap, thus the transfer and release process of DOX can be sensitively detected by FRET signal.

As shown in Figure 2b, after incubating with GQDs@GE11/DOX/CDDP nanoprobe for 0.5 h, red fluorescent signal (DOX FRET channel) was showed at the border area of CNE‐2 cells, indicating that the nanocarriers began to enter CNE‐2 cells. At this initial stage, the fluorescence of GQDs was basically in the “OFF” state (GQDs channel), while DOX was “ON”, indicating that DOX molecules were mainly accumulated on the surface of GQDs. After 4 h of incubation, the blue fluorescence (GQDs channel) in the cytoplasm of cells in the “ON” state was significantly increased due to the DOX molecules falling off the carrier. After 8 h incubation, strong red fluorescence could be noticed in the cell (DOX 485 ex channel), while the FRET signal (DOX FRET channel) was reduced, indicating that most DOX molecules were released from the carrier and accumulate in the nucleus. The results showed that the nanoprobe could sensitively detect the intracellular drug release process at the level of a single cancer cell, which may be an effective tool to detect the drug control release process.

### 3.5 Cytotoxicity of GQDs@GE11

**FIGURE 3:**
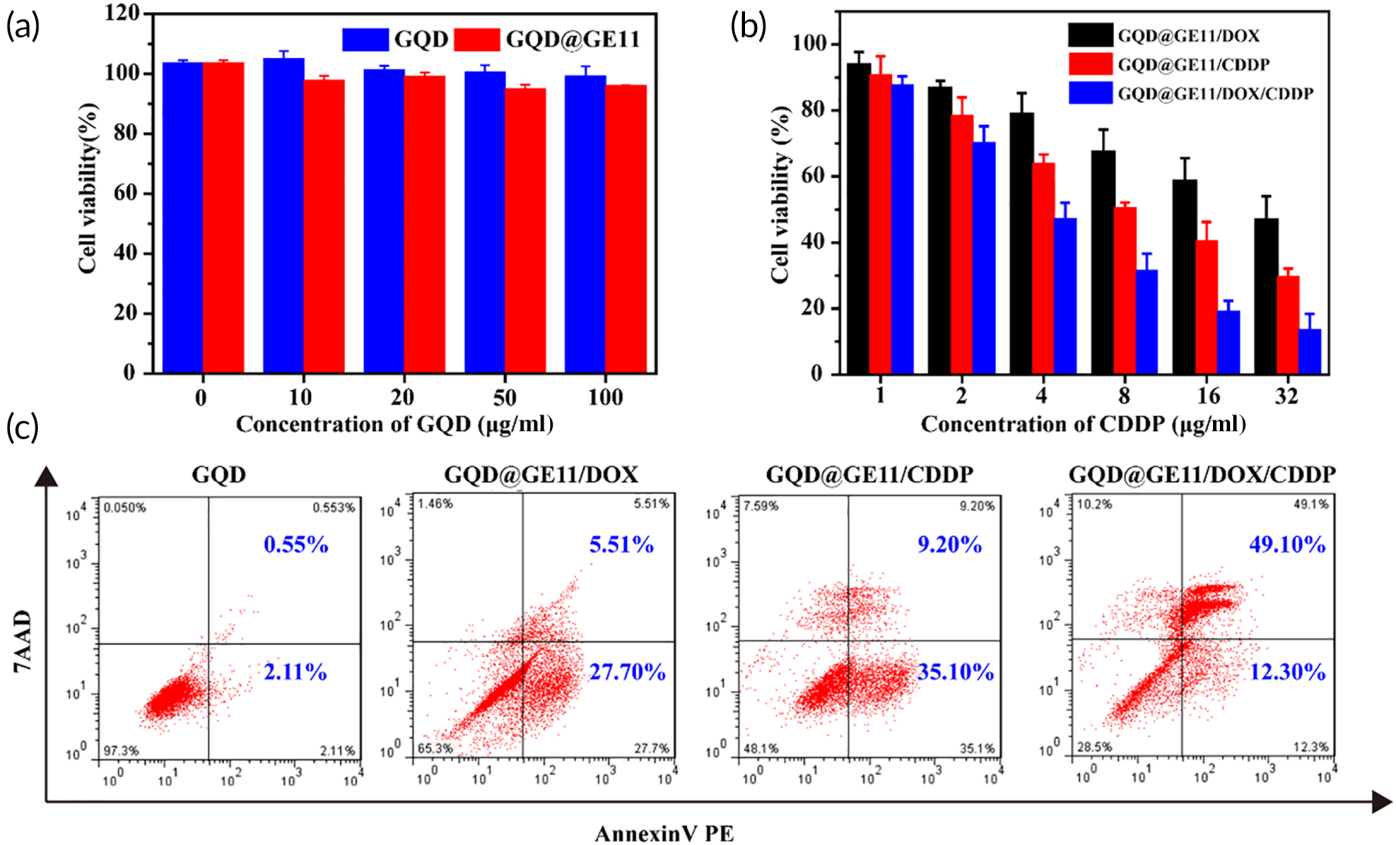
(a) In vitro cytotoxicity of GQDs or GQDs@GE11 with different concentrations on CNE‐2 cells over an evaluation period of 24 h. (b) Cell viability of CNE‐2 cells pretreated by GQDs@GE11/DOX, GQDs@GE11/DOX and GQDs@GE11/DOX /CDDP with different concentrations. (c) Apoptosis analysis by flow cytometry after CNE‐2 cells incubated with various formulations using Annexin‐V PE and 7‐ADD dual‐staining method

Low cytotoxicity is the basic requirement for nanoprobe's in vivo application. Here, we conducted an in vitro cytotoxicity assay by co‐culture CNE‐2 cells with different concentration (0, 10, 20, 50, and 100 μg/ml) of GQD and GQD@GE11 nanoprobes, and the cell viability was tested by CCK‐8 assay. As shown in Figure 3a, the cell viability of all groups exceeded 90%, indicating that the GQD and GQD@GE11 nanoprobes at all concentrations almost shown no toxicity to CNE‐2 cells. These results showed that GQDs and GQDs@GE11 nanoprobes had good cell compatibility.

### 3.6 Inhibition of cell proliferation

The CCK‐8 method further confirmed the feasibility of GQD@GE11/DOX/CDDP to inhibit the proliferation of CNE‐2 cells in vitro. After 24 h co‐cultured with CNE‐2 cells, the in vitro cytotoxicity of GQDs@GE11/DOX, GQDs@GE11/CDDP, and GQDs@GE11/DOX/CDDP nanoprobes was determined. Almost all experimental groups had inhibitory effects on CNE‐2 cells, and the inhibitory effect was concentration‐dependent (Figure 3b). In addition, the cytotoxicity of GQDs@GE11/DOX/CDDP nanoprobe was significantly higher than that of GQDs@GE11/DOX and GQDs@GE11/CDDP nanoprobes, which could be explained by the effect of combination therapy over single drug therapy.

As previous studies reported, the antitumor effects of DOX and CDDP both depend on their ability to interact with DNA.^43^ DOX, as an anthracycline topoisomerase II inhibitor, could partially hinder the effective repair of DNA damaged by alkylating agents, and had been observed to increase the efficacy of CDDP on many tumor cells lines, proving that the drug combination has better the tumor suppressor effect. For patients with advanced nasopharyngeal cancer, the enhanced chemotherapy effect is of great significance for killing cancer cells and increasing the survival rate of patients. The in vitro inhibitory effect on CNE‐2 cells proved that the GQDs@GE11/CDDP nanoprobe would help the treatment of nasopharyngeal carcinoma.

### 3.7 In vitro apoptosis assessments

Apoptosis was also conducted to evaluate the killing effect of GQDs@GE11/DOX/CDDP nanoprobe on CNE‐2 cells.

The CNE‐2 cells after treatment with different nanoprobes were stained with Annexin V‐PE and 7‐ADD. The apoptotic cell population after nanoprobes treatment was quantitatively analyzed with flow cytometry. As shown in Figure 3c, cells treated with blank GQDs@GE11 nanoprobe showed negligible apoptosis, indicating that the nanoprobe as drug carrier did not induce apoptosis in CNE‐2 cells. Both DOX and CDDP could significantly induce CNE‐2 cells apoptosis. The cells processed with GQDs@GE11/DOX, GQDs@GE11/CDDP, and GQDs@GE11/DOX/CDDP nanoprobes showed the 33.21%, 44.3%, and 61.4% of apoptosis, respectively. Compared with GQDs@GE11/DOX nanoprobe and GQDs@GE11/CDDP nanoprobe, the apoptosis rate of CNE‐2 cell treatment with GQDs@GE11/DOX/CDDP nanoprobe was significantly higher, indicating the synergistic treatment strategy could induce tumor cell apoptosis well, which was consist with the in vitro cell proliferate inhibition assay.

### 3.8 In vivo antitumor efficacy of GQDs@GE11/CDDP/DOX nanoprobe

Encouraged by the enhanced CNE‐2 cells killing effect of GQDs@GE11/CDDP/DOX nanoprobe in vitro, the nanoprobe has high potential to treat nasopharyngeal carcinoma. Thus, we assessed the antitumor effect in the transplanted tumor mouse model. PBS treated was selected as the control group for better comparison.

**FIGURE 4:**
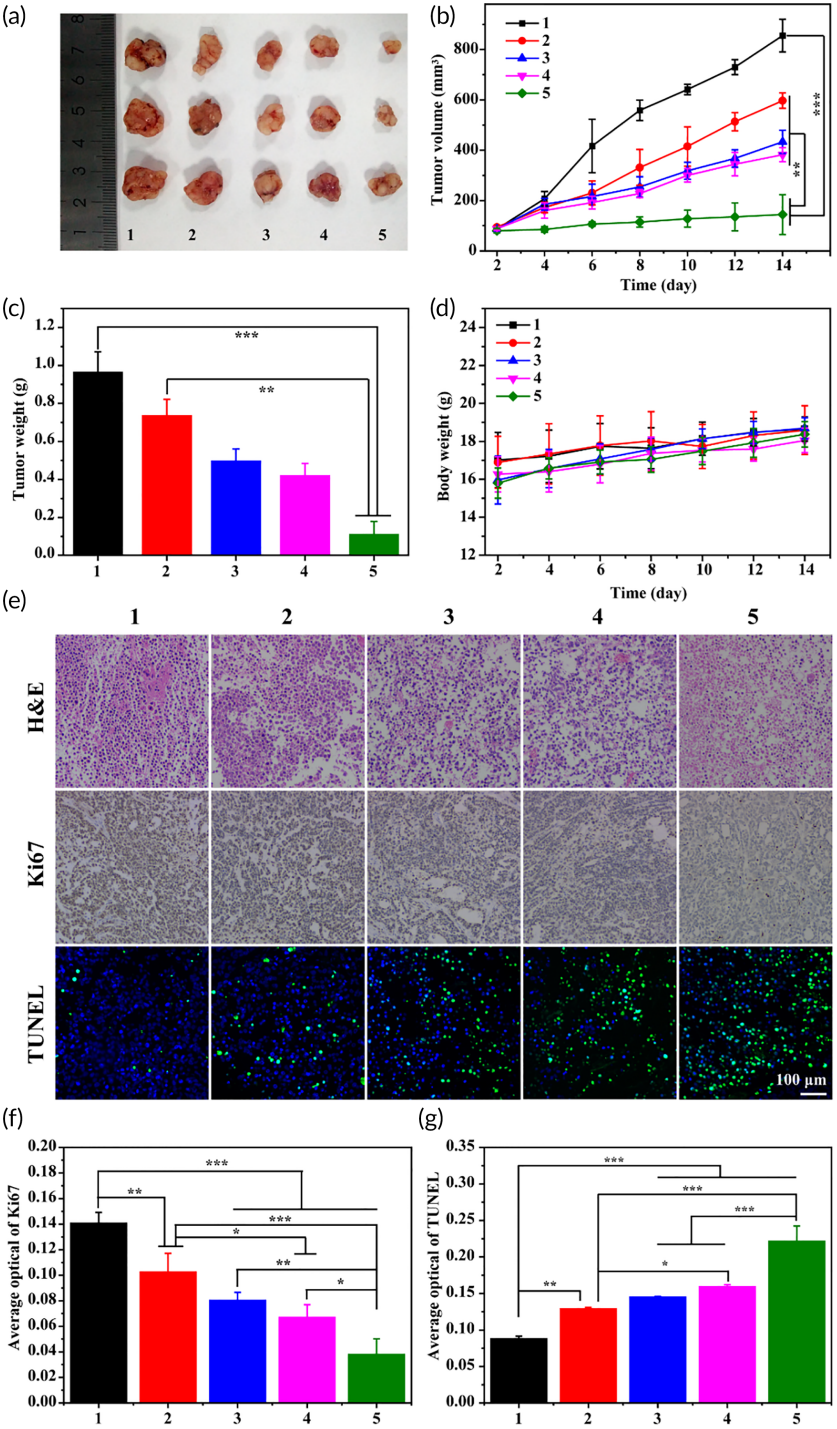
(a) Representative image of CNE‐2 tumors at the 14th day. (b) In vivo tumor growth curves of CNE‐2 tumor‐bearing mice treated with different formulations. (c) The tumor weights excised from different groups after 14 days treatment. (d) Body weight changes of mice treated with different formulations during the treatment. (e) Immunohistochemical analyses of H&E, TUNEL, CD31, and Ki67 for CNE‐2 tumor tissues after the last treatment with different formulations in vivo (200×). (f) Quantitative analysis of Ki67 immunohistochemical positive area. (g) Quantitative analysis of TUNEL in tumor sites. (1, PBS; 2, GQDs@GE11/DOX; 3, GQDs@GE11/CDDP; 4, GQDs@/DOX/CDDP; 5, GQDs@GE11/DOX/CDDP). One‐way ANOVA and Tukey post hoc analysis. *p < 0.05; **p < 0.01; and ***p < 0.001

As shown in Figure 4b, the tumor growth in the control group could not be blocked, and the volumes of tumors increased rapidly to an average volume of about 1000 mm^3^ within 14 days. Other four groups treated with GQDs@GE11/DOX, GQDs@GE11/CDDP, GQDs/CDDP/DOX, and GQDs@GE11/CDDP/DOX nanoprobes all showed effective tumor suppression effects. It is worth noting that the antitumor effect of GQDs@GE11/CDDP/DOX nanoprobe was superior to that of GQDs@GE11/DOX, GQDs@GE11/CDDP, and GQDs/CDDP/DOX nanoprobes, and tumor volume was only 144 mm^3^. This may be due to the fact that targeting polypeptide GE11 modified synergistic combination of nanolevel drugs enhanced drug accumulation and retention at the tumor site.

The tumor image and tumor weight data after 14 days are shown in Figure 4a and c. Tumor weight treated by GQDs@GE11/CDDP/DOX nanoprobes was almost 10 times lighter than control groups and nearly four times lighter than untargeted GQDs/CDDP/DOX nanoprobes. These results further indicated that DOX and CDDP have effective antitumor effects on CNE‐2 tumors in vivo, and the co‐drug targeting delivery system of GQDs@GE11/CDDP/DOX showed much better tumor suppressor effect among them. During the in vivo experiment, the changes in the weight of the mice were recorded and considered to be a key factor for reflecting the safety and side effects of the single formula used. As shown in Figure 4d, the body weight of mice in each group increased, which means GQDs@GE11/CDDP/DOX nanoprobe was safe for mice in the process of tumor treatment in vivo.

### 3.9 Histologic and immunohistochemical analysis

Through the histopathological analysis of H&E‐stained CNE‐2 tumor sections, the antitumor efficacy of co‐delivery GQDs@GE11/CDDP/DOX nanoprobe was further evaluated. As shown in Figure 4e, the results highly support the tumor suppression data. The tumor cells treated with PBS have a complete structure and more chromatin, indicating that the tumor grows rapidly. However, the other groups treated with DOX and CDDP showed reduced tumor cells and enlarged intracellular spaces to varying degrees, suggesting that these groups showed effective treatment responses to tumors. It was particularly noteworthy that GQDs@GE11/CDDP/DOX treated tumor cells with the widest intracellular space and the least number of tumor cells, indicating that GQDs@GE11/CDDP/DOX nanoprobe had the best therapeutic effect.

TUNEL immunofluorescent staining examination was further conducted to evaluate the cell apoptosis effects of different treatments (Figure 4e) and quantitative analysis is shown in Figure 4g. Similarly, GQDs@GE11/CDDP/DOX induced the highest proportion of apoptosis‐positive tumor cells, confirming its strongest antitumor effect in vivo. The antitumor efficacy of each group was detected by the expression of Ki67 (Figure 4f). Compared with other treatment groups, the GQDs@GE11/CDDP/DOX nanoprobe was the most effective in reducing tumor cell proliferation, resulting in the lowest Ki67 expression in CNE‐2 cells. Taken together, the GQDs@GE11/CDDP/DOX nanoprobe has the best antitumor effect in vivo.

### 3.10 In vivo fluorescent imaging

**FIGURE 5:**
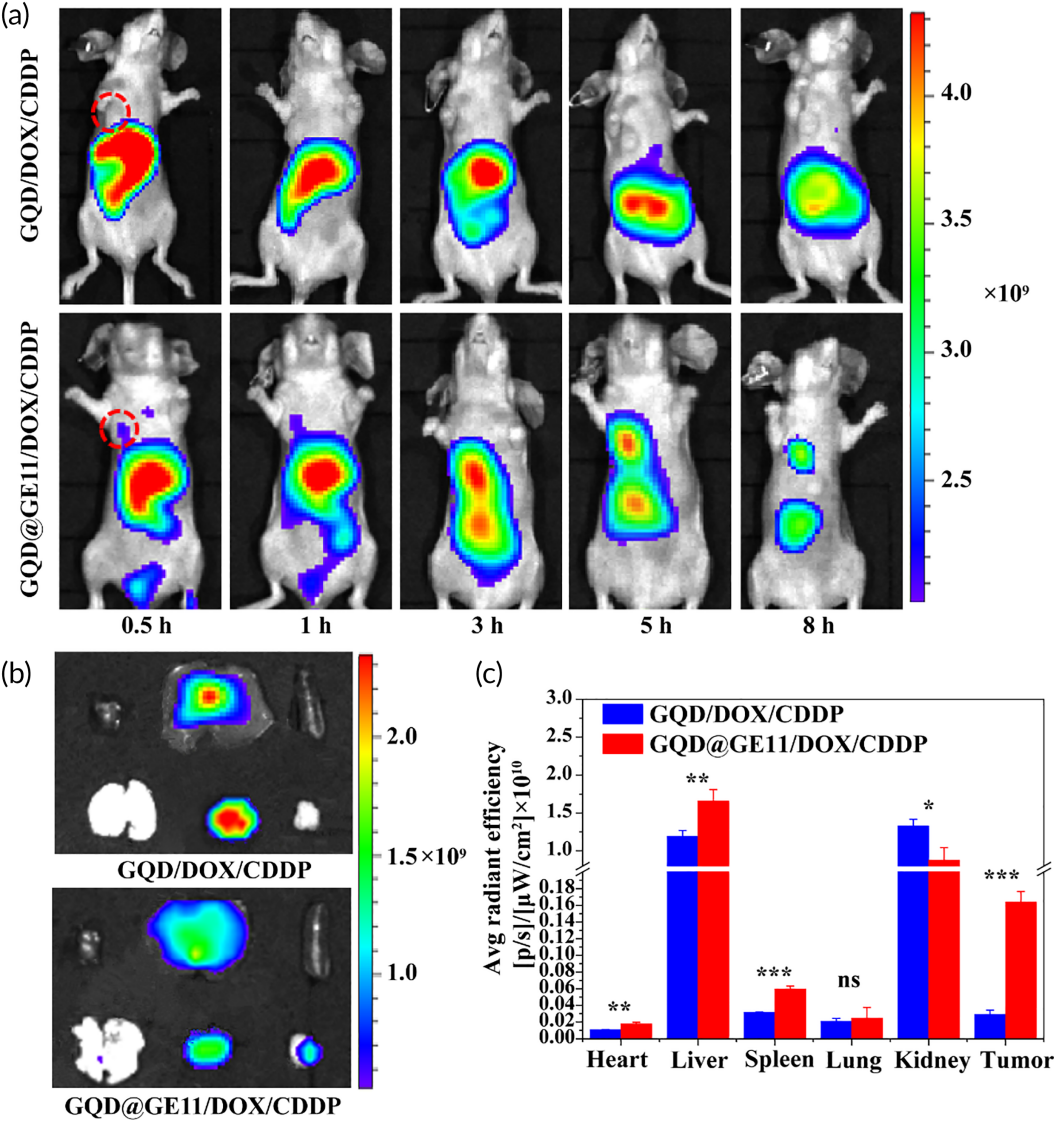
(a) In vivo fluorescence imaging of tumor‐bearing nude mice after intravenous injection of GQDs@GE11/DOX/CDDP and GQDs/DOX/CDDP complexes at different times. (b) GQDs@GE11/DOX/CDDP and GQDs/DOX/CDDP treatments for 8 h of main organ fluorescence image. (c) Quantitative analysis of fluorescence intensity of main organs and tumor. One‐way ANOVA and Tukey post hoc analysis. *p < 0.05; **p < 0.01; and ***p < 0.001

To further evaluate the optical imaging and tumor enrichment of GQDs in mice, GQDs@GE11/DOX/CDDP and GQDs/DOX/CDDP were injected into Balb/c nude mice through the tail vein. Figure 5a showed the time‐dependent optical image in mice after injection of the material (GQDs is 10 mg/kg dose). After intravenous injection of GQDs@GE11/DOX/CDDP for 1 h, obvious fluorescence signal was seen in the tumor, but negligible fluorescent signal was seen in the tumor after GQDs/DOX/CDDP treatment. The fluorescence intensity of tumor tissue collected after GQDs@GE11/DOX/CDDP treatment was significantly stronger than that of GQDs/DOX/CDDP treatment (Figure 5b), further confirming the high targeting efficiency of GQDs@GE11/DOX/CDDP.

### 3.11 Biocompatibility evaluation

**FIGURE 6:**
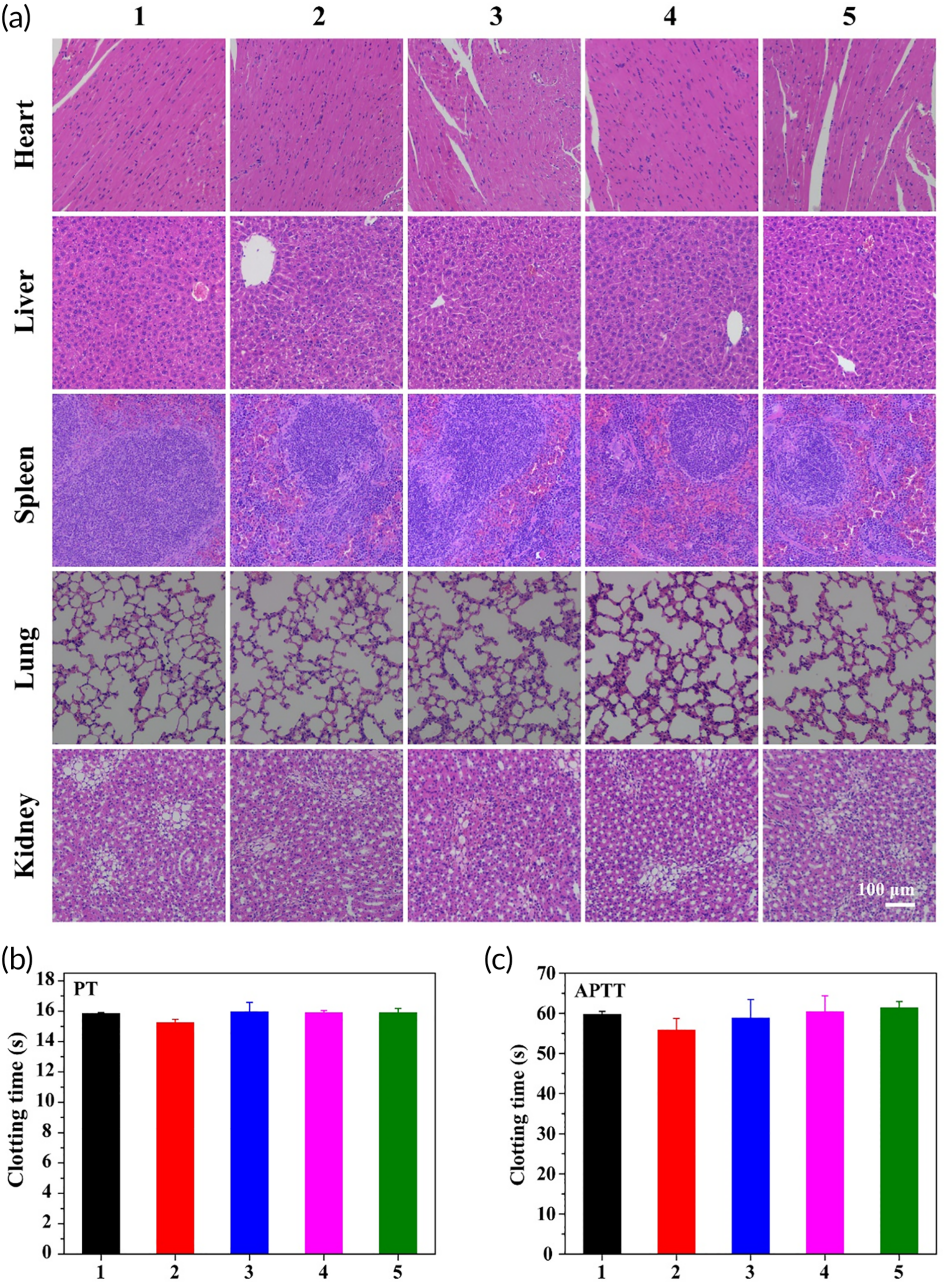
(a) Histologic assessments of major organs in mice (×200) treated with different formulations. (b) After 14 days of treatment, the serum biochemical results of mice. (1, PBS; 2, GQDs@GE11/DOX; 3, GQDs@GE11/CDDP; 4, GQDs@/DOX/CDDP; 5, GQDs@GE11/DOX/CDDP)

Biocompatibility was a necessary condition for the safe application of materials in nanomedicine. Therefore, we studied the in vivo toxicity of different treatment groups to the main organs and blood of mice. H&E images of the main organs of the mice after 14 days of different treatments are shown in Figure 6a. There was no obvious damage to the tissue morphology and structure of the organs in each treatment group. Blood chemistry indexes of each group were also detected. Compared with the PBS control group, the blood parameters of the mice in the treatment group did not change significantly (Figure 6b, c). To sum up, GQDs@GE11 nanoprobe is safety and necessity for the treatment of nasopharyngeal carcinoma.

### 3.12 Hemolysis assay in vitro

**FIGURE 7:**
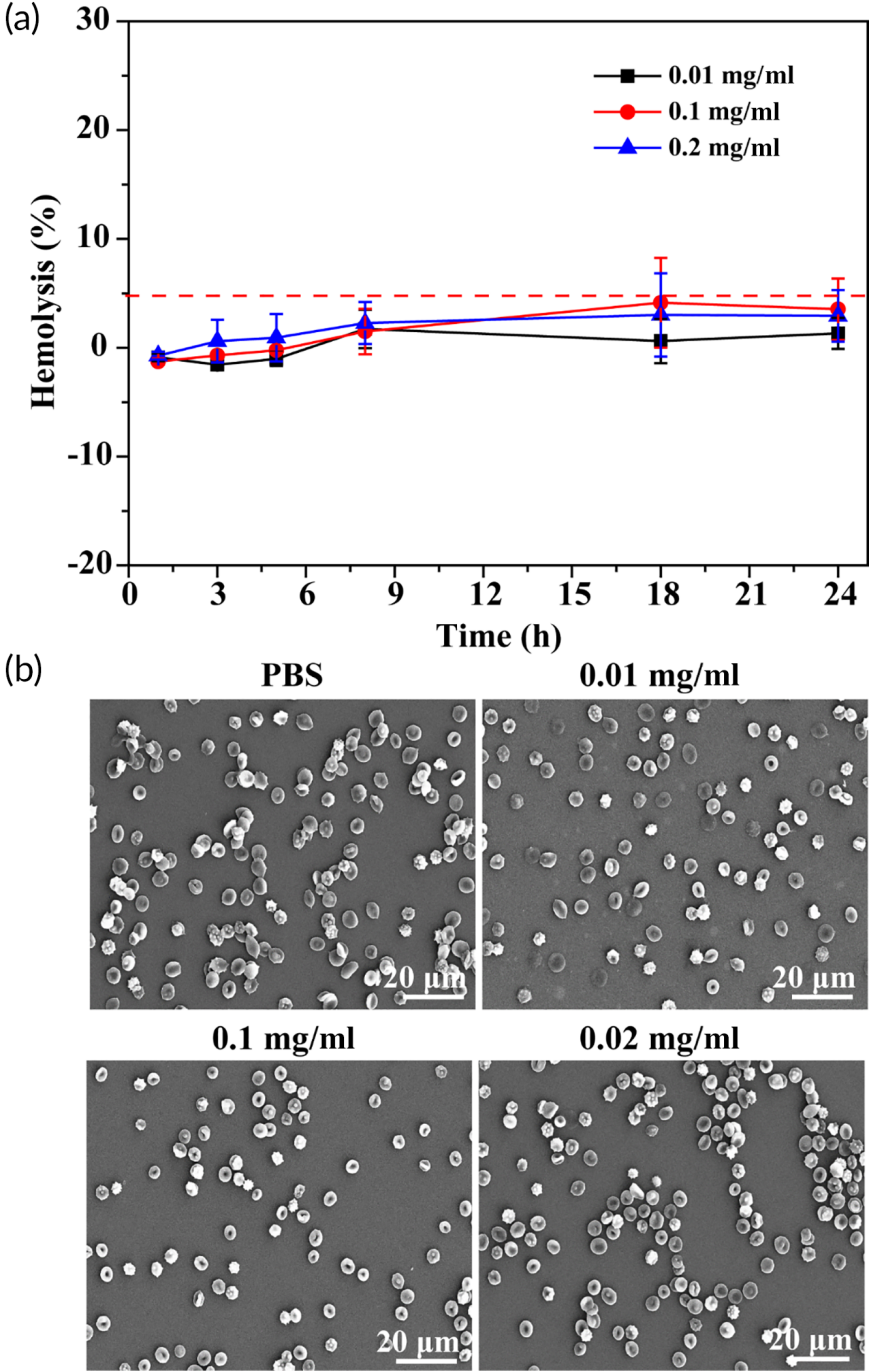
(a) Effect of GQDs@GE11with different concentrations on the hemolysis. (b) Effect of GQDs@GE11 with different concentrations on the aggregation and morphology of RBCs

The interaction between cells and materials first occurs on the cell membrane,^44^ thus affecting its structure and function in many ways. Hemolysis refers to the release of hemoglobin from RBCs, indicating that the integrity of the RBC membrane is disturbed. Therefore, it reflects the interaction of biological materials with RBC membranes. Figure 7a showed the percentage of hemolysis of RBC exposed to different concentrations of GQDs@GE11 and different incubation times. The GQDs@GE11 had no significant hemolysis at the highest 0.2 mg/ml, indicating that the RBC membrane had no detectable interference.

### 3.13 Morphology of RBCs

In humans, mature RBCs are elastic biconcave disks, lacking a nucleus and most organelles; they are very sensitive to membrane active substances. In this study, the aggregation and morphological changes of RBC by GQDs@GE11 was used to examine by scanning electron microscopy (SEM) (Figure 7b). Compared with the PBS group, GQDs@GE11 did not cause RBC aggregation or morphological changes at concentrations of 0.01 and 0.1 mg/ml. But in the presence of 0.2 mg/ml GQDs@GE11, RBCs were aggregate though shape does not change. It proved that GQDs@GE11 had good blood compatibility within a specific concentration range.

## 4 CONCLUSION

In summary, we designed an innovative combination therapy platform based on GQDs, which has the functions of drug delivery and in vivo imaging. By monitoring the changes in the fluorescence spectrum signals of tumor cells, the cellular uptake of nanoprobes and the release of intracellular drug molecules were successfully monitored. In addition, by combining with the targeting polypeptide GE11, GQDs@GE11/DOX/CDDP was enriched at the tumor site, enhancing the therapeutic effect of the drug. GQDs@GE11 showed good cell compatibility within the concentration range studied. The present study faces some limitations that future experiments should consider. For example, this research only studied the targeted endocytosis of nanoprobes to CNE‐2 cells and their distribution in major organs, however, distribution of nanoprobes in other organs and the mechanism of entering the body have not been studied. These need to be further studied in subsequent research to improve the clinical translation prospects of nanoprobes. To sum up, nanoprobes based on GQDs have drug molecular tracking functions and are expected to become a platform for precise diagnosis and treatment of targeted cancer.

## CONFLICT OF INTEREST

The authors declare no conflict of interest.

## AUTHOR CONTRIBUTIONS

**Chaosheng Yu:** Data curation (equal); methodology (lead); software (equal); writing – original draft (lead). **Zhen Long:** Conceptualization (equal); formal analysis (lead); investigation (lead); visualization (equal). **Qianhui Qiu:** Data curation (equal); project administration (equal); supervision (equal). **Fang Liu:** Resources (equal); software (equal); validation (equal). **Yiming Xu:** Validation (equal); visualization (equal); writing – original draft (supporting). **Tao Zhang:** Formal analysis (equal); project administration (equal); resources (equal). **Rui Guo:** Resources (equal); supervision (equal). **Shuaijun Chen:** Conceptualization (equal); funding acquisition (lead); resources (equal); writing – review and editing (lead).

### PEER REVIEW

The peer review history for this article is available at https://publons.com/publon/10.1002/btm2.10270.

## Data Availability

corresponding author. The data are not publicly available due to privacy or ethical restrictions.

## References

[1] Ding Y , Xiao X , Zeng L , et al. Platinum‐crosslinking polymeric nanoparticle for synergetic chemoradiotherapy of nasopharyngeal carcinoma. Bioactive Mater. 2021;6(12):4707‐4716. 10.1016/j.bioactmat.2021.05.010 PMC8164009 34095627

[2] Ding R‐B , Chen P , Rajendran BK , et al. Molecular landscape and subtype‐specific therapeutic response of nasopharyngeal carcinoma revealed by integrative pharmacogenomics. Nat Commun. 2021;12(1):3046. 34031426 10.1038/s41467-021-23379-3 PMC8144567

[3] Chen Y‐P , Chan ATC , le QT , Blanchard P , Sun Y , Ma J . Nasopharyngeal carcinoma. The Lancet. 2019;394(10192):64‐80. 10.1016/S0140-6736(19)30956-0 31178151

[4] Blanchard P , Lee A , Marguet S , et al. Chemotherapy and radiotherapy in nasopharyngeal carcinoma: an update of the MAC‐NPC meta‐analysis. Lancet Oncol. 2015;16(6):645‐655. 25957714 10.1016/S1470-2045(15)70126-9

[5] Lee A , Chow JCH , Lee NY . Treatment Deescalation strategies for nasopharyngeal cancer: a review. JAMA Oncol. 2021;7(3):445‐453. 10.1001/jamaoncol.2020.6154 33355642

[6] de Ruysscher D , Niedermann G , Burnet NG , Siva S , Lee AWM , Hegi‐Johnson F . Radiotherapy toxicity. Nat Rev Dis Prim. 2019;5:13. 30792503 10.1038/s41572-019-0064-5

[7] Chan L , Chen X , Gao P , et al. Coordination‐driven enhancement of Radiosensitization by black phosphorus via regulating tumor metabolism. ACS Nano. 2021;15(2):3047‐3060. 33507069 10.1021/acsnano.0c09454

[8] You Y , He L , Ma B , Chen T . High‐drug‐loading mesoporous silica Nanorods with reduced toxicity for precise cancer therapy against nasopharyngeal carcinoma. Adv Funct Mater. 2017;27(42):1703313.

[9] You Y , Zhao Z , He L , et al. Long‐term oxygen storage Nanosystem for near‐infrared light‐triggered oxygen supplies to antagonize hypoxia‐induced therapeutic resistance in nasopharyngeal carcinoma. Adv Funct Mater. 2020;30(27):2002369.

[10] Ma PA et al. Inorganic nanocarriers for platinum drug delivery. Mater Today. 2015;18(10):554‐564.

[11] Ghawanmeh AA , Ali GAM , Algarni H , Sarkar SM , Chong KF . Graphene oxide‐based hydrogels as a nanocarrier for anticancer drug delivery. Nano Research. 2019;12(5):973‐990.

[12] Martín C , Ruiz A , Keshavan S , et al. A biodegradable multifunctional graphene oxide platform for targeted cancer therapy. Adv Funct Mater. 2019;29(39):1901761.

[13] Yang X , Zhang X , Liu Z , Ma Y , Huang Y , Chen Y . High‐efficiency loading and controlled release of doxorubicin hydrochloride on graphene oxide. Jphyschemc. 2008;112(45):17554‐17558.

[14] Karimi E , Jaafar HZ , Ahmad S . Antifungal, anti‐inflammatory and cytotoxicity activities of three varieties of labisia pumila benth: from microwave obtained extracts. BMC Complement Altern Med. 2013;13(1):1‐10. 23347830 10.1186/1472-6882-13-20 PMC3608971

[15] Zhang B , Wang Y , Zhai G . Biomedical applications of the graphene‐based materials. Materials Ence & Engineering C. 2016;61(Apr.):953‐964. 10.1016/j.msec.2015.12.073 26838925

[16] Qi Z , Wu Z , Ning L , et al. Advanced review of graphene‐based nanomaterials in drug delivery systems: synthesis, modification, toxicity and application. Materials Science & Engineering C. 2017;77:1363‐1375. 28532014 10.1016/j.msec.2017.03.196

[17] Huang J , Huang W , Zhang Z , et al. Highly uniform synthesis of selenium nanoparticles with EGFR targeting and tumor microenvironment‐responsive ability for simultaneous diagnosis and therapy of nasopharyngeal carcinoma. ACS Appl Mater Interfaces. 2019;11(12):11177‐11193. 30821437 10.1021/acsami.8b22678

[18] Luo Y , Wang J , Wang F , et al. Foxq1 promotes metastasis of nasopharyngeal carcinoma by inducing vasculogenic mimicry via the EGFR signaling pathway. Cell Death Dis. 2021;12(5):411. 33875643 10.1038/s41419-021-03674-z PMC8055972

[19] Liu Y , Zhao Y , Luo H , Liu F , Wu YM . Construction of EGFR peptide gefitinib/quantum dots long circulating polymeric liposomes for treatment and detection of nasopharyngeal carcinoma. Biochem Biophys Res Commun. 2017;490(2):141‐146. 28595906 10.1016/j.bbrc.2017.06.011

[20] Shi J , Chan C , Pang Y , et al. A fluorescence resonance energy transfer (FRET) biosensor based on graphene quantum dots (GQDs) and gold nanoparticles (AuNPs) for the detection of mecA gene sequence of Staphylococcus aureus. Biosens Bioelectron. 2015;67:595‐600. 25288044 10.1016/j.bios.2014.09.059

[21] Su X , Chan C , Shi J , et al. A graphene quantum dot@Fe3O4@SiO2 based nanoprobe for drug delivery sensing and dual‐modal fluorescence and MRI imaging in cancer cells. Biosens Bioelectron. 2016;92:489‐495. 27839733 10.1016/j.bios.2016.10.076

[22] Nasrollahi F , Koh YR , Chen P , Varshosaz J , Khodadadi AA , Lim S . Targeting graphene quantum dots to epidermal growth factor receptor for delivery of cisplatin and cellular imaging. Mater Sci Eng C Mater Biol Appl. 2018;94:247‐257. 30423706 10.1016/j.msec.2018.09.020

[23] Nurunnabi M , Khatun Z , Huh KM , et al. In vivo biodistribution and toxicology of Carboxylated graphene quantum dots. ACS Nano. 2013;7(8):6858‐6867. 23829293 10.1021/nn402043c

[24] Liu W , Pan Y , Zhong Y , et al. A multifunctional aminated UiO‐67 metal‐organic framework for enhancing antitumor cytotoxicity through bimodal drug delivery. Chem Eng J. 2021;412:127899.

[25] Xing E , Du Y , Yin J , et al. Multi‐functional Nanodrug based on a three‐dimensional framework for targeted photo‐chemo synergetic cancer therapy. Adv Healthc Mater. 2021;10(8):2001874. 10.1002/adhm.202001874 33448142

[26] Lv X , Cao X , Xia WX , et al. Induction chemotherapy with lobaplatin and fluorouracil versus cisplatin and fluorouracil followed by chemoradiotherapy in patients with stage III–IVB nasopharyngeal carcinoma: an open‐label, non‐inferiority, randomised, controlled, phase 3 trial. Lancet Oncol. 2021;22(5):716‐726. 33857411 10.1016/S1470-2045(21)00075-9

[27] Modjtahedi H , Komurasaki T , Toyoda H , Dean C . Anti‐EGFR monoclonal antibodies which act as EGF, TGF alpha, HB‐EGF and BTC antagonists block the binding of epiregulin to EGFR‐expressing tumours. Int J Cancer. 1998;75(2):310‐316. 9462724 10.1002/(sici)1097-0215(19980119)75:2<310::aid-ijc22>3.0.co;2-f

[28] Di Fiore PP et al. Overexpression of the human EGF receptor confers an EGF‐dependent transformed phenotype to NIH 3T3 cells. Cell. 1987;51(6):1063‐1070. 3500791 10.1016/0092-8674(87)90592-7

[29] Park EJ , Min HY , Chung HJ , et al. Down‐regulation of c‐Src/EGFR‐mediated signaling activation is involved in the honokiol‐induced cell cycle arrest and apoptosis in MDA‐MB‐231 human breast cancer cells. Cancer Lett. 2009;277(2):133‐140. 19135778 10.1016/j.canlet.2008.11.029

[30] Chen J , Ding J , Xu W , et al. Receptor and microenvironment dual‐recognizable Nanogel for targeted chemotherapy of highly metastatic malignancy. Nano Lett. 2017;17:4526‐4533. 28644032 10.1021/acs.nanolett.7b02129

[31] He T , Li D , Yang Y , et al. Mesomeric configuration makes polyleucine micelle an optimal nanocarrier. Biomater Sci. 2016;4(5):814‐818. 26876808 10.1039/c6bm00022c

[32] Hummers WS , Offeman RE . Preparation of graphitic oxide. Am Chem Soc. 1958;208:1334‐1339.

[33] Zhu S , Zhang J , Qiao C , et al. Strongly green‐photoluminescent graphene quantum dots for bioimaging applications. Chem Commun. 2011;47(24):6858‐6860. 10.1039/c1cc11122a 21584323

[34] Ghorbani M , Abdizadeh H , Golobostanfard MR . Reduction of graphene oxide via modified hydrothermal method. Procedia Mater Sci. 2015;11:326‐330.

[35] Sinha R , Purkayastha P . Daunomycin delivery by ultrasmall graphene quantum dots to DNA duplexes: understanding the dynamics by resonance energy transfer. J Mater Chem B. 2020;8(42):9756‐9763. 33021304 10.1039/d0tb01831g

[36] Wang C , Chen Y , Xu Z , et al. Fabrication and characterization of novel cRGD modified graphene quantum dots for chemo‐photothermal combination therapy. Sens Actuators B. 2020;309:127732.

[37] Sawy AM , Barhoum A , Abdel Gaber SA , et al. Insights of doxorubicin loaded graphene quantum dots: synthesis, DFT drug interactions, and cytotoxicity. Mater Sci Eng C. 2021;122:111921. 10.1016/j.msec.2021.111921 33641914

[38] Qiu J , Zhang R , Li J , et al. Fluorescent graphene quantum dots as traceable, pH‐sensitive drug delivery systems. Int J Nanomedicine. 2015;10:6709‐6724. 26604747 10.2147/IJN.S91864 PMC4630193

[39] Yu SM , Gonzalez‐Moragas L , Milla M , et al. Bio‐identity and fate of albumin‐coated SPIONs evaluated in cells and by the C. elegans model. Acta Biomater. 2016;43:348‐357. 27427227 10.1016/j.actbio.2016.07.024

[40] Zhou C , Xia Y , Wei Y , et al. GE11 peptide‐installed chimaeric polymersomes tailor‐made for high‐efficiency EGFR‐targeted protein therapy of orthotopic hepatocellular carcinoma. Acta Biomater. 2020;113:512‐521. 32562803 10.1016/j.actbio.2020.06.020

[41] Brinkman AM , Chen G , Wang Y , et al. Aminoflavone‐loaded EGFR‐targeted unimolecular micelle nanoparticles exhibit anti‐cancer effects in triple negative breast cancer. Biomaterials. 2016;101:20‐31. 27267625 10.1016/j.biomaterials.2016.05.041 PMC5030715

[42] Ma Y , Zhao Y , Bejjanki NK , et al. Nanoclustered cascaded enzymes for targeted tumor starvation and deoxygenation‐activated chemotherapy without systemic toxicity. ACS Nano. 2019;13(8):8890‐8902. 31291092 10.1021/acsnano.9b02466

[43] Liu J , Wei T , Zhao J , et al. Multifunctional aptamer‐based nanoparticles for targeted drug delivery to circumvent cancer resistance. Biomaterials. 2016;91:44‐56. 26994877 10.1016/j.biomaterials.2016.03.013

[44] Elferink CJ , Ge NL , Levine A . Maximal aryl hydrocarbon receptor activity depends on an interaction with the retinoblastoma protein. Mol Pharmacol. 2001;59(4):664‐673. 11259609 10.1124/mol.59.4.664

